# Synchronous Bilateral Benign Phyllodes Tumor of the Breast in a 32-year-old Woman

**DOI:** 10.4021/wjon2010.01.1204

**Published:** 2010-02-01

**Authors:** Amel Trabelsi, Soumaya Ben Abdelkrim, Faten Hammedi, Wassila Sahraoui, Atef Ben Abdelkader, Badreddine Sriha, Moncef Mokni

**Affiliations:** a Department of Pathology, Farhat Hached Hospital, Sousse, Tunisia; b Department of Gynecology, Farhat Hached Hospital, Sousse, Tunisia

**Keywords:** Breast, Synchronous bilateral tumors, Phyllodes tumor

## Abstract

Bilateral phyllodes tumors are distinctly uncommon. As some previous reports have described, most of them are malignant and asynchronous. We report a new case of bilateral synchronous phyllodes tumor in a 32-year-old women. Both tumors were classified as benign after large bilateral excision. No tumor recurrence was noted during the 10 months follow-up.

## Introduction

Phyllodes tumor, initially fully characterized by Johannes Muller in 1838 [1], constitutes 0.3 to 1% of all breast neoplasms. Bilateral phyllodes tumors are extremely rare [2]. We describe a new case of synchronous bilateral phyllodes tumor in a 32-year-old woman.

## Case Report

**Figure 1:**
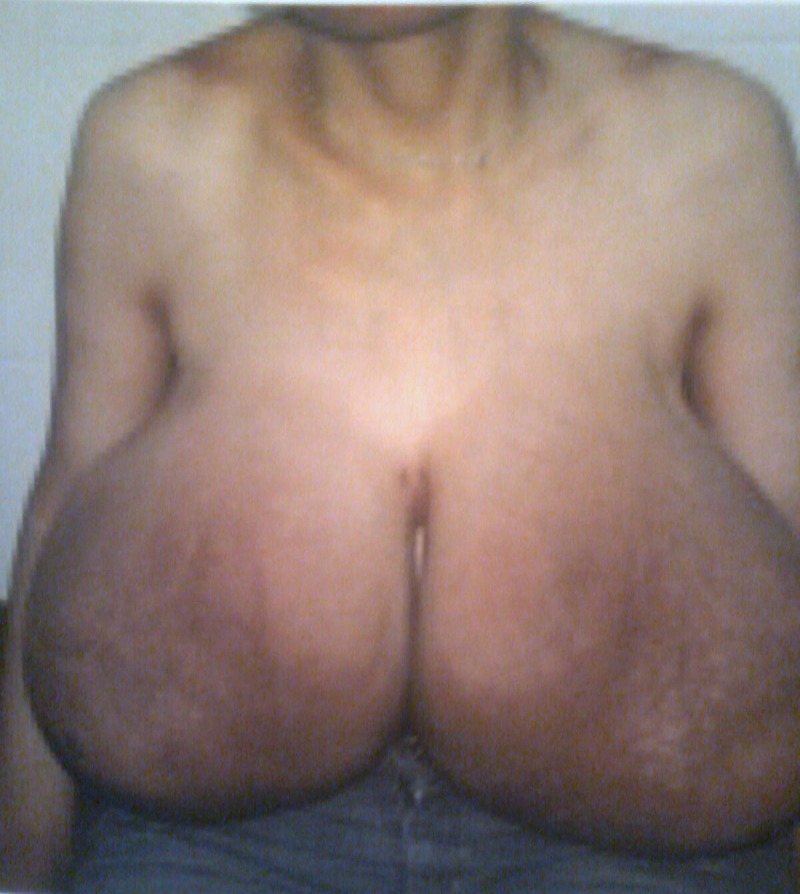
Breast bilateral asymmetric enlargement.

**Figure 2:**
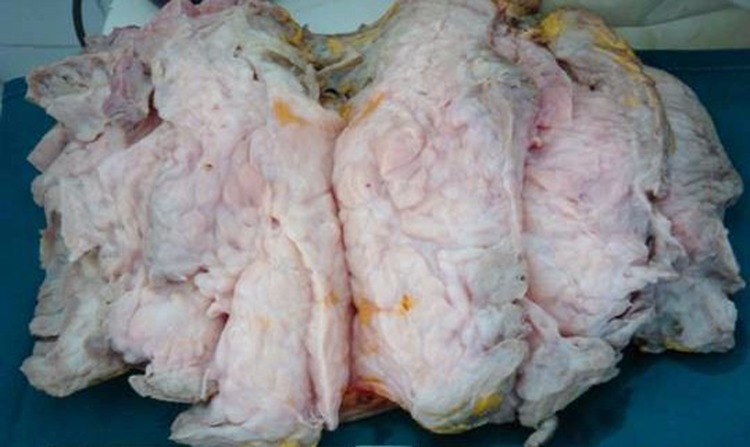
Macroscopic appearance of the tumor.

**Figure 3:**
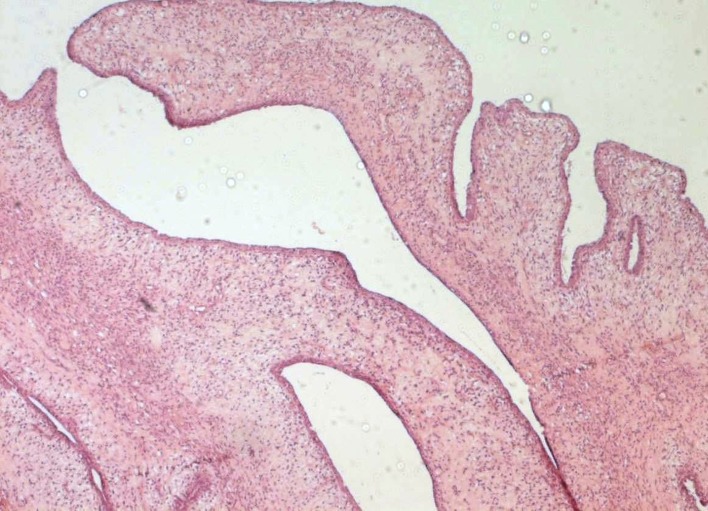
Benign phyllodes tumor (magnification x 100).

A 32-year-old woman, with uneventful medical and surgical history, presented with breast bilateral asymmetric enlargement (Fig. 1) of three months duration. Physical examination showed a large mass occupying the entire right breast of 30 cm in diameter. The left breast showed a bilobate mass measuring 25 cm. Radiologic evaluation included only ultrasonography and showed smooth bordered heterogeneous hypoechoic masses. Excisional biopsies and frozen-section analysis assessed the fibroepithelial nature of both tumors. Treatment consisted in complete surgical removal of the two masses. Macroscopically, the left tumor and the right one presented as white, firm in consistency, well demarcated, unencapsulated masses of 38 and 57 cm in diameter respectively (Fig. 2). Histological findings demonstrated, in both tumors, a biphasic neoplasm consisting of stromal component lacking atypia and showing a low mitotic index (3 mitoses per 10 high power fields). There were clefts lined by a bilayered regular epithelium (Fig. 3). The tumors showed a sharp limitation but were not encapsulated. These morphological features were consistent with the diagnosis of bilateral benign phyllodes tumor.

## Discussion

Phyllodes tumors, also called cystosarcoma, are classified as benign, borderline and malignant. This subclassification is based on microscopic findings, including stromal cellularity, cellular pleomorphism, mitotic activity, margins’ appearance and stromal distribution [1]. Unlike fibroadenoma, bilateral phyllodes tumor is extremely rare [2-5]. Most of the reported cases were asynchronous with a free interval ranging from 6 months to 8.5 years [2]. Bilateral phyllodes tumors are usually malignant [2, 5, 6]. We believe that we have described here the first case of benign bilateral synchronous phyllodes tumor. Like ipsilateral phyllodes tumor, surgical removal is the mainstay of treatment.
